# Advances and Future Perspectives on Chitosan-Based Hydrogels for Bone Regeneration in Periodontitis-Associated Defects

**DOI:** 10.3390/ijms27219489

**Published:** 2026-10-24

**Authors:** Zixuan Li, Yuanyuan Yang, Yujie Zheng, Xuelian Su, Jing Wen, Hengyan Liang, Manli Guo

**Affiliations:** 1 Key Laboratory of Stomatology of State Ethnic Affairs Commission, Northwest Minzu University, Lanzhou 730030, China; p232115370@stu.xbmu.edu.cn (Z.L.); p232115130@stu.xbmu.edu.cn (Y.Y.); p232115124@stu.xbmu.edu.cn (Y.Z.); 284122153@xbmu.edu.cn (J.W.); 285122145@xbmu.edu.cn (H.L.); guoml@xbmu.edu.cn (M.G.); 2 Key Laboratory of Oral Diseases of Gansu Province, Northwest Minzu University, Lanzhou 730030, China; 3 Medical Department, Northwest Minzu University, Lanzhou 730030, China

**Keywords:** CS-based hydrogels, periodontitis, alveolar bone defects, immune regulation, biomimetic scaffold, stimuli-responsive controlled release, bone regeneration

## Abstract

Periodontitis-triggered alveolar bone loss is an irreversible pathological lesion that remains challenging to regenerate in clinical practice. Functionally modified chitosan (CS) hydrogels combine favorable biocompatibility, tunable biodegradability, intrinsic antibacterial activity, and immunomodulatory properties, rendering them promising scaffolds for the repair of inflammatory bone defects. This review summarizes advances over the past five years in the development of functionalized CS hydrogels for periodontitis-associated alveolar bone regeneration. We categorize and compare four mainstream modification strategies for CS: biomimetic structural design, inflammatory immunoregulation, stimuli-responsive controlled release, and mechanical microenvironment adaptation. Beyond hydrogels, chemically modified CS in diverse forms (membranes, bulk scaffolds, micro/nanoparticles, and surface coatings) is also discussed. Furthermore, we comprehensively elaborate on the core regenerative mechanisms of modified CS hydrogels, including antibacterial defense, immune microenvironment remodeling, osteogenic activation, and angiogenesis–osteogenesis coupling. Unlike most previous reviews covering CS biomaterials, the present work specifically focuses on the challenges of bone regeneration under periodontitis-related inflammatory conditions, with a small set of other bone regeneration studies included as supporting references. It examines the key limitations of current investigations—particularly those concerning mechanical matching, sustained anti-inflammatory efficacy, and clinical translatability—and outlines targeted research directions involving structural optimization, multistage cascade regulation, and personalized clinical translation. Overall, this review provides a theoretical framework and practical design strategies for developing high-performance CS-based hydrogels toward periodontal bone defect regeneration.

## 1. Introduction

Periodontitis is characterized by persistent microbial infection, immune dysregulation, and progressive irreversible alveolar bone resorption that ultimately leads to tooth mobility and tooth loss [1]. Alveolar bone loss induced by periodontitis represents irreversible pathological damage. While guided tissue regeneration (GTR) yields moderate clinical benefits, functional restoration remains hindered by two primary obstacles: compromised biomechanical integrity of residual bone and persistent dysregulation of the local immunoinflammatory microenvironment [2,3]. Moreover, the dynamic mechanical loading associated with mastication places stringent demands on the mechanical adaptability of regenerative scaffolds. Conventional biomaterials often fail to withstand these complex mechanical conditions, increasing the risk of scaffold deformation or collapse and, consequently, impaired tissue repair [4,5,6]. Therefore, given the multifaceted challenges of alveolar bone defect repair—including inflammation control, tissue regeneration, and mechanical support—the design of multifunctional scaffolds that integrate anti-inflammatory and osteogenic bioactivities, adapt to the complex mechanical microenvironment of periodontal tissues, modulate local immune responses, and promote vascularization constitutes a promising tissue engineering strategy for functional alveolar bone regeneration.

As a natural cationic polysaccharide, CS exhibits excellent biocompatibility, controllable degradability, inherent antibacterial properties, and facile modifiability, making it a leading candidate material for repairing inflammatory bone defects [7,8]. CS-based hydrogels can be fabricated by blending CS as the primary matrix with other functional components, followed by crosslinking. These hydrogels are capable of constructing biomimetic extracellular microenvironments to regulate immune cell polarization and osteogenic differentiation of stem cells, thereby enabling concurrent inflammation resolution and bone regeneration and conferring unique advantages for repairing periodontitis-associated defects [7,9,10]. Recent advances have further endowed CS-based hydrogels with stimuli-responsive, antioxidant, and ion-releasing functions to target specific pathological pathways in periodontal disease [11,12].

Existing reviews on CS hydrogels have notable critical gaps. First, most prior reviews address general bone tissue engineering applications while overlooking the unique immuno-mechanical coupling within the periodontal microenvironment; they lack targeted discussions on the distinct inflammatory microenvironment of periodontitis and adaptive scaffold design [13,14]. Second, the current literature lacks systematic classification and comparative analysis of spatiotemporal synergy across different modification strategies [15,16,17]. Finally, few reviews independently and profoundly analyze the core technical bottlenecks restricting the clinical translation of CS hydrogels in periodontal regeneration.

Against this backdrop, the present review specifically focuses on periodontitis-associated alveolar bone regeneration. It highlights the pathological features of periodontal inflammation and summarizes CS hydrogel modification strategies tailored for inflammatory bone repair. A systematic classification of optimization approaches is presented, accompanied by a comparative analysis of their merits, limitations, and applicable scenarios. Key challenges related to mechanical matching, anti-inflammatory stability, controlled release, and clinical translation are examined, and multidimensional future research directions are proposed. Section-specific commentaries are also included to evaluate the strengths and weaknesses of current technologies, aiming to provide theoretical support and practical design considerations for CS-based scaffolds in periodontal bone regeneration.

### Literature Search Strategy

This review retrieved recent publications from PubMed, Google Scholar, and Web of Science, using search terms including “bone regeneration”, “inflammatory periodontal alveolar bone regeneration”, “chitosan”, “modified chitosan”, and “biomimetic scaffolds”. Initial screening was performed by titles and abstracts to exclude irrelevant, duplicate, and review articles, as well as conference abstracts and studies with incomplete experimental data. The final included original studies centered on CS-based materials, focusing on their preparation, modification, and characterization. These studies investigated the therapeutic efficacy and regenerative mechanisms of CS materials for bone defect repair, especially within inflamed periodontal microenvironments, alongside their prospects for clinical translation.

## 2. Classification and Horizontal Comparison of Mainstream CS-Based Hydrogel Optimization Strategies

The rational design of CS-based hydrogels for periodontal regeneration must simultaneously address multiple challenges inherent to the inflammatory microenvironment, including structural disintegration under mechanical loading, dysregulated immune responses, pathological bacterial colonization, and impaired spatiotemporal coordination of tissue repair. To tackle these challenges, we categorize biomaterials for periodontitis-associated inflammatory alveolar bone regeneration into four functional dimensions: (i) the structural level—how material architecture recapitulates the native extracellular matrix; (ii) the biological signaling level—how the material communicates with and directs the local immune and cellular milieu; (iii) the temporal control level—how therapeutic agents are released in response to specific pathological cues; and (iv) the biomechanical level—how the material adapts to and modulates physical forces within regenerating tissue.

It is important to emphasize that these four dimensions are not mutually exclusive categories but rather complementary analytical perspectives. A single material system may concurrently embody features from multiple dimensions. For instance, a 3D-printed scaffold incorporating anti-inflammatory nanoparticles with pH-responsive release behavior simultaneously achieves structural design, immune modulation, and stimuli-responsive delivery. Each strategy has distinct technical principles, performance characteristics, and application boundaries. This section systematically summarizes the four optimization strategies and compares their technical characteristics, clarifying their differences and strengthening the systematicness and comprehensiveness of this review (Table 1). Remaining study-level evidence is compiled in Table 2 to display material composition, payloads, and evidence tiers.

**Table 2:** Representative studies on CS-based materials and related constructs for periodontal/alveolar bone regeneration. Studies are categorized into three groups: periodontal/alveolar bone defect models (T1), non-periodontal oral/maxillofacial bone defect models (T2), and non-oral in vivo models (T3). Parameters not reported in the original literature are indicated as “NR” (not reported).

Evidence Tier	Composition	Crosslinking	Bioactive Payload	Structure	Release Profile	Model	Defect	Outcome	Limitation	Ref.
T1	Aligned CS/oxidized chondroitin sulfate hydrogel	NR	None	Aligned, anisotropic porous architecture	None	Rat periodontal defect	Periodontal defect	Oriented periodontal-ligament fibers with alveolar bone regeneration	Pore size was not quantified; long-term multi-tissue outcomes were not established	[18]
T1	Thermosensitive CS/β-glycerophosphate/gelatin hydrogel	Thermosensitive gelation	Aspirin + EPO	NR	Sequential early aspirin/later EPO release	Rat ligature-induced periodontitis	Periodontitis-associated bone defect	Reduced inflammation and improved bone healing	Two-stage release; payload-free attribution remains limited	[24]
T1	CS/poloxamer 407/crosslinked HA hydrogel	Crosslinked HA network	Oxygen + sEVs	Nanoporous	Spatiotemporal oxygen/sEV release	Periodontal bone-defect model	Periodontal bone defect	Reduced anaerobic bacterial load and promoted alveolar bone regeneration	Multi-component system; independent matrix contribution not isolated	[28]
T1	pH-responsive CS/β-glycerophosphate hydrogel	pH-responsive composite	Minocycline hydrochloride	NR	pH-dependent antibiotic release	Sprague-Dawley rat ligature-induced periodontitis	Periodontitis-associated bone defect	Reduced NLRP3 activation/inflammation and promoted alveolar bone regeneration	No matrix-only control	[29]
T1	Fucoidan–CS hydrogel	NR	Fucoidan	NR	NR	Randomized clinical trial; 40 patients	Two-wall intrabony periodontal defects	Greater radiographic bone fill, but no significant differences in several clinical endpoints	9-month reported follow-up; internally inconsistent reporting between abstract and results	[30]
T1	β-tricalcium phosphate/CS/SBA-15 scaffold	NR	Metformin	3D porous scaffold	Metformin-loaded scaffold	Rat periodontitis model	Periodontal bone defect	Increased alveolar bone regeneration over 12 weeks	Metformin and CS contributions not separated	[31]
T1	CS particles, low- and high-molecular-weight	Particle formulation	None	Particles	NR	Randomized clinical trial; 18 defects	Three-wall intrabony defects	No between-group differences in clinical or radiographic outcomes	Very small groups; no evidence of superiority	[32]
T1	CS nanoparticles with or without melatonin	Nanoparticle formulation	Melatonin	Nanoparticles	Melatonin-loaded nanoparticles	Three-arm randomized clinical trial; 67 defects	Intrabony periodontal defects	Greater defect-height reduction with melatonin-loaded nanoparticles; CS-only arm included	No hard-tissue histology; carrier and payload effects remain formulation-specific	[33]
T1	Oscillating CS device ± CS gel	Mechanical device	CS gel adjunct	Device	NR	Randomized clinical trial	Residual pockets in stage III–IV periodontitis	Greater probing-depth reduction and pocket closure reported for adjunctive treatment	Clinical attachment level was not a main endpoint; corresponding author disclosed company equity	[34]
T1	Oscillating CS brush + ultrasonic debridement	Mechanical debridement	CS brush	Brush/device	NR	Split-mouth randomized clinical trial	Peri-implant/periodontal inflammatory site	No between-group difference in key clinical parameters; difference reported only for bleeding on probing	No bone-regeneration endpoint	[35]
T2	EPO-loaded CS/β-glycerophosphate/gelatin hydrogel	Thermosensitive gelation	EPO	NR	Sequential/controlled EPO delivery	Rabbit maxillary sinus floor-elevation model	Maxillary sinus floor elevation	Increased new bone volume and mineral density	Oral but non-periodontal model	[36]
T3	Quaternized CS coating on Ti-6Al-4V	Covalent surface anchoring	H_2_S	Implant coating	H_2_S remained below antibacterial threshold	Rat subcutaneous implantation ± MRSA	Subcutaneous implant model	Antibacterial activity, epithelial/fibroblast adhesion, M2 polarization	Non-oral model; no periodontal bone-defect validation	[22]
T3	Tannic-acid-modified CS/alginate microspheres	Microsphere formulation	None	Microsphere system	NR	Rat tibial critical-size defect	Non-oral bone defect	Reduced osteoclastogenesis and promoted BMSC osteogenesis	Not a hydrogel; non-periodontal model	[37]

### 2.1. Biomimetic Structural Optimization of CS-Based Hydrogels

Porous structure and spatial morphology govern the cell infiltration, nutrient transport, and tissue ingrowth capacity of hydrogel scaffolds. Unmodified pure cross-linked CS hydrogels typically form compact network structures with small pore sizes and low porosity, failing to meet the spatial demands of alveolar bone regeneration. Current structural optimization strategies include pore-size regulation, double-network construction, nanocomposite modification, and layered gradient biomimetic design. Porous CS scaffolds designed for bone tissue engineering have been reviewed with respect to the relationship between pore architecture and osteogenesis as well as vascularization [38]. Interconnected macroporous CS-based hydrogels overcome nutrient diffusion limitations seen in dense hydrogels, providing favorable conditions for the adhesion and proliferation of periodontal ligament stem cells (PDLSCs) and bone marrow mesenchymal stem cells (BMSCs) [18,39].

Nanomodification using sepiolite nanotubes, lignocellulosic nanofibers, and nano-hydroxyapatite (nHA) effectively improves the structural stability and osteoinductivity of scaffolds [38,40,41]. A wood-derived strontium-loaded CS hydrogel has been evaluated in calvarial defect models [41]. Nanoporous hydrogels composed of CS, poloxamer 407 (P407), and crosslinked hyaluronic acid have been engineered to enable spatiotemporally controlled release of oxygen and small extracellular vesicles (sEVs). These hydrogels reduce anaerobic bacterial burden while promoting alveolar bone regeneration in periodontal bone defect models [28]. Nanoporous CS-based scaffolds have been proposed as extracellular matrix mimics that modulate cell migration, adhesion, and osteogenic differentiation; this evidence has been summarized from the perspective of scaffold architecture [38]. In silk fibroin (SF)/CS composite porous scaffolds, porosity gradually decreases and pore-size uniformity deteriorates as the chitosan proportion increases. The SF80/CS20 scaffold exhibits optimal pore characteristics, supports good cell adhesion and viability, and facilitates the repair of rabbit tibial bone defects [42]. Aligned porous CS/oxidized chondroitin hydrogel scaffolds seeded with PDLSCs and gingival-derived mesenchymal stem cells (GMSCs) can induce ordered arrangement of periodontal ligament fibers and alveolar bone regeneration, showing promise for periodontal tissue regeneration in rat models [18].

### 2.2. Immunomodulatory and Anti-Inflammatory Functional Optimization

**Figure 1:**
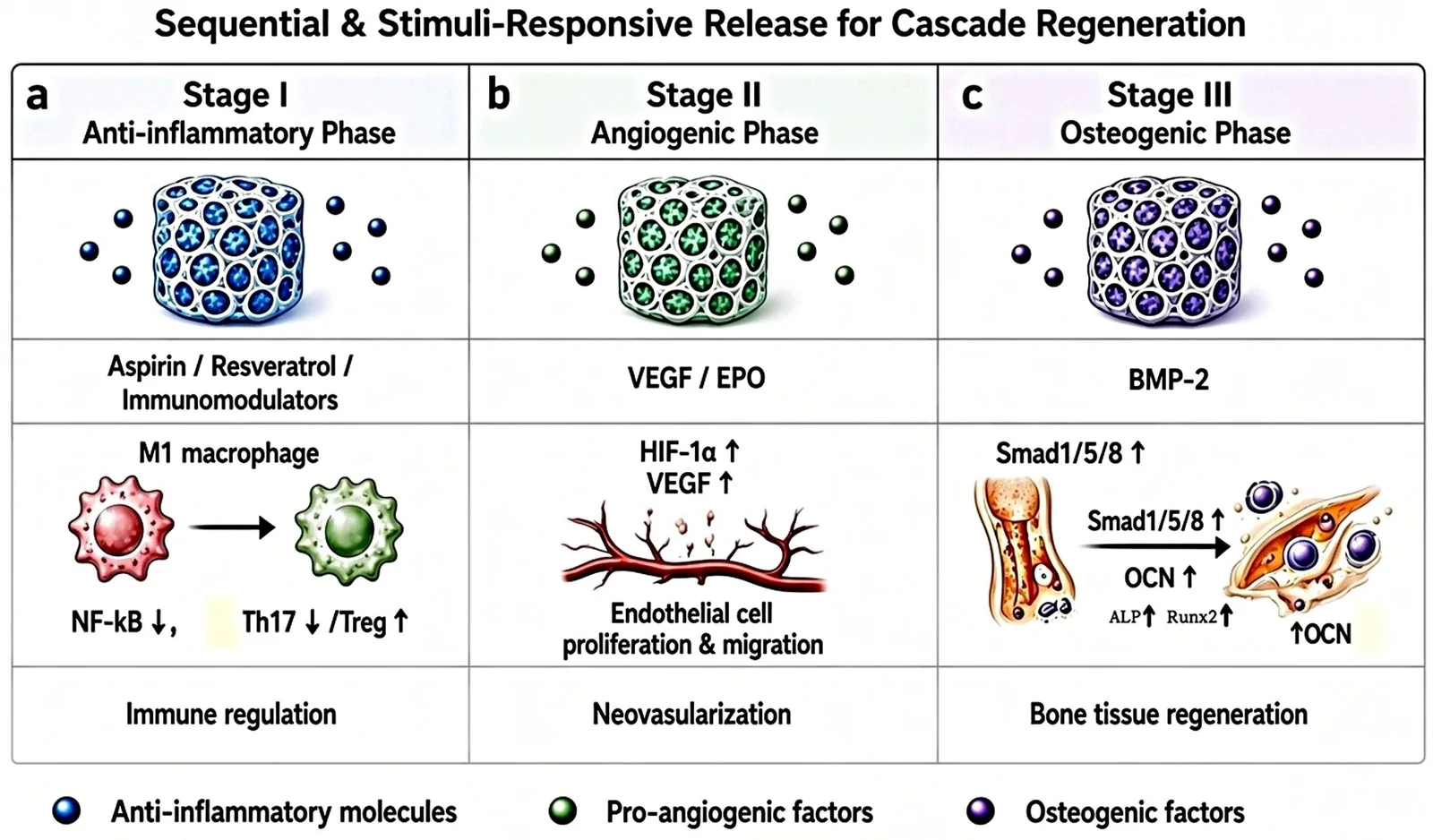
Schematic illustration of sequential biomolecule release and associated tissue repair processes. (**a**) Anti-inflammatory phase: M1-to-M2 macrophage polarization and Th17/Treg balance restoration. (**b**) EPO release stimulates endothelial cell proliferation and angiogenesis. (**c**) BMP-2 release promotes osteogenesis and bone regeneration.

Persistent chronic inflammation constitutes the primary barrier to successful periodontal bone regeneration. It arises from dysregulated host immune responses to pathogenic bacteria, which necessitates precise modulation of inflammation to achieve favorable periodontal regeneration. CS hydrogels exhibit intrinsic immunomodulatory capacity to modulate the polarization of macrophages, dendritic cells (DCs) and T lymphocytes. Accordingly, CS-based hydrogels serve as versatile platforms for spatiotemporally controlled delivery of diverse bioactive agents including growth factors, antimicrobials and immunoregulatory molecules, which can simultaneously address infection, inflammation and tissue repair [29] (Figure 1). CS loaded with anti-inflammatory compounds (minocycline hydrochloride, resveratrol, quercetin, embelin) or bioactive metal ions (strontium, copper) can synergistically improve the regulation of the inflammatory microenvironment [29,41]. Implantation of the multi-crosslinked chitosan-P407-PNIPAM scaffold hydrogel (A/CQ-ML@Gel) in an SD rat periodontitis model markedly inhibits *Porphyromonas gingivalis (P. gingivalis)*. Through programmed release of quercetin microemulsion and phenethyl caffeate from CQ-ML, the hydrogel achieves robust inflammatory regulation and promotes osteogenic differentiation of PDLSCs [43]. Meanwhile, the cationic nature of CS disrupts the membrane integrity of periodontal pathogens (e.g., *P. gingivalis*), yielding combined antibacterial and anti-inflammatory effects [38].

Accumulating experimental evidence demonstrates that functionally modified CS-based hydrogels exert beneficial effects on the repair of inflammatory bone defects. For example, oxidized starch/CS hydrogels loaded with ampicillin sodium enable sustained controlled delivery over 29 h to mitigate local inflammation [44], whereas thermosensitive CS/β-glycerophosphate hydrogels co-loaded with BMP-7 and ornidazole exert synergistic antibacterial, anti-inflammatory, and osteogenic effects in canine periodontitis models [45]. Comparable staged-delivery systems co-releasing aspirin (ASA) and erythropoietin (EPO) further validate this paradigm: early ASA suppresses COX-2 activity, osteoclast maturation, and inflammatory bone resorption, while sequentially released EPO enhances angiogenesis, cell proliferation, and tissue regeneration [24]. A broad spectrum of additional bioactive agents, including the NF-κB inhibitor embelin [46], geniposide [47], metronidazole [48], antimicrobial peptides, and fucoidan [30], have also been incorporated into CS-based scaffolds to achieve sustained local delivery, simultaneously eradicating pathogens, resolving inflammation, and triggering regenerative cascades. Collectively, these multifunctional CS-based hydrogel platforms represent an attractive therapeutic strategy to counteract inflammation-suppressed bone formation in periodontitis.

In addition, CS derivatives hold triple synergistic potential combining antibacterial, anti-inflammatory, and pro-regenerative effects within the periodontal inflammatory microenvironment. Their inherent cationic property endows them with broad-spectrum antibacterial activity, and chemical modification further strengthens targeted elimination of periodontal pathogens. As a typical chemically modified CS variant, quaternized CS carries pH-independent charge; hence, its antibacterial activity does not rely on amino-group protonation. Quaternized CS coatings on titanium implant surfaces can kill *Staphylococcus aureus* (*S. aureus*), *Escherichia coli* (*E. coli*), *P. gingivalis*, and *Fusobacterium nucleatum* (*F. nucleatum*), and inhibit biofilm formation on implant surfaces [22]. Nevertheless, available studies only confirm that quaternized chitosan inhibits alveolar bone resorption, while its capacity to promote alveolar bone regeneration has not yet been reported [49]. In contrast, multiple in vivo studies using alternative CS derivatives in rat periodontitis models have demonstrated concurrent anti-inflammatory and osteogenic benefits. One investigation adopted carboxymethyl chitosan (CMCS)-modified copper sulfide nanoparticles; another used curcumin-loaded CMCS/HA composites; and the third applied sulfated chitosan surface-functionalized CuS nanomaterials [50,51,52]. All these formulations effectively alleviate local inflammatory responses and promote alveolar bone regeneration in rat periodontitis models [50,51,52].

Immunomodulatory anti-inflammatory modification represents the core functional strategy enabling CS hydrogels to adapt to periodontal inflammatory lesions, fundamentally mitigating inflammation-induced bone resorption. Even so, current immune modulation is mostly static and lacks dynamic responsiveness to real-time shifts in the inflammatory microenvironment. The relatively short sustained-release window of anti-inflammatory factors also limits long-term maintenance of immune homeostasis in chronic inflammatory defects. Finally, inflammation models vary substantially: ligature-induced periodontitis, high-fat-diet-associated periodontitis, and surgically created defects carry unequal inflammatory burdens.

### 2.3. Stimuli-Responsive Sequential Release System Optimization

Alveolar bone repair proceeds as a sequential process: pathogen clearance and inflammation resolution dominate the early phase, whereas osteogenic differentiation and bone mineralization prevail at later stages. Conventional hydrogels suffer from uncontrolled burst release and mismatched release kinetics and therefore fail to match this staged repair cascade. In contrast, stimuli-responsive CS hydrogels adopt pH-, temperature-, and reactive oxygen species (ROS)-responsive dual or multimodal designs to achieve spatiotemporal control of payload delivery, which is critical for coordinating inflammation resolution and tissue regeneration (Figure 1). A PDA-modified hydroxybutyl CS hydrogel; loaded with near-infrared-responsive microspheres rapidly releases ASA followed by sustained BMP-2 release. The fast ASA release suppresses early inflammation, and sustained BMP-2 release supports later-stage osteogenesis in rat calvarial defects [23]. Similarly, a thermosensitive CS/β-glycerophosphate/gelatin hydrogel was designed for sequential delivery of ASA and subsequently erythropoietin (EPO) in a rat ligature-induced periodontitis model, yielding improved therapeutic outcomes via timed release of the two agents [24]. In another work, a biguanidine-functionalized injectable CS hydrogel enables local sequential delivery of VEGF and BMP-2 to promote angiogenesis and osteogenic differentiation of dental pulp stem cells, thereby accelerating bone regeneration [53]. In a rat periodontitis model, a layered scaffold implanted into bone defects sequentially releases ASA from the outer layer to reprogram the inflammatory microenvironment, followed by osteogenic growth peptide (OGP) from the inner core to drive alveolar bone regeneration [54].

Architectural engineering constitutes a vital strategy for tuning hydrogel release profiles. Injectable thermosensitive CS-based hydrogels with 3D porous networks co-deliver BMP-2 and phototherapeutic agents; incorporation of H_2_O_2_ enhances photodynamic antibacterial efficacy, and this system reduces periodontal bone loss in a mouse ligature and *P. gingivalis*-induced periodontitis model [55]. A dual-crosslinked CS/Pluronic F-127/tripolyphosphate composite releases bone morphogenetic protein-9 (BMP-9) for over 36 days at pH 7.4 (and over 25 days at pH 6.0), though this study addressed femoral head osteonecrosis [56]. In a study on deacetylated CS coatings on polycaprolactone (PCL) microporous scaffolds, coating deacetylation degree dictated innate immune bioactivity and differentially stimulated angiogenesis and osteogenesis in rabbit knee osteochondral defects [57]. CS–aloe vera scaffolds carrying sEVs together with BMP-2 and PLGA microsphere-encapsulated histatin-5 doubled the in vitro osteogenic differentiation level compared with pure CS scaffolds [58].

Thus, optimized release kinetics and stimuli-responsive delivery act synergistically with local microenvironment regulation. Combined exploitation of stimulus responsiveness, architectural engineering, and composite modification endows smart CS platforms with refined spatiotemporal delivery of biomolecules, offering promising candidates for the therapy of periodontal inflammatory bone defects. Compared with traditional single-release scaffolds, stimuli-responsive sequential delivery represents a key advance for coordinating inflammation resolution and bone regeneration. However, the complex and heterogeneous periodontal microenvironment may weaken material responsiveness and destabilize drug release. Furthermore, most current systems only support two-stage release and lack sophisticated multistage regulation covering the entire bone repair cascade. Accordingly, multi-stimuli-responsive carriers should be prioritized in future investigations to improve delivery precision and accommodate the complicated microenvironments of bone lesions. Lastly, many payloads consist of potent drugs or growth factors, whose doses are selected for biological efficacy rather than safety; therefore, safety validation is required for the achieved payload concentrations and release rates.

### 2.4. Mechanical Microenvironment Adaptive Optimization

Alveolar bone is continuously subjected to compressive, tensile, and shear mechanical stimulation during mastication [59]. Nevertheless, pure CS hydrogels exhibit low modulus, poor toughness, and rapid degradation, rendering them prone to structural collapse and loss of supportive function under dynamic oral mechanical conditions. An ideal regenerative scaffold must not only possess favorable biocompatibility and tunable degradability but also provide biomechanically matched support: preserving spatial integrity at the defect site while delivering physiologically relevant mechanical cues to resident cells. To overcome these drawbacks, multiple strategies—including polymer blending (e.g., CMC, SA, silk fibroin (SF)), covalent or physical cross-linking, and incorporation of nano-bioactive ceramics—have been developed to substantially enhance the compressive strength and structural stability of CS-based hydrogels. This allows their modulus to be tuned to match that of native alveolar cancellous bone [60].

The properties of CS-based hydrogels and their derivatives also show dose-dependent behavior. For example, when 10–30 wt% HA is incorporated, the stiffness and mechanical strength of CS-based scaffolds increase markedly with rising HA concentration [26]; tannic acid (TA)-crosslinked quaternized CS hydrogels possess tunable elastic moduli ranging from 110 to 1000 Pa. Increasing TA concentration promotes polymer chain entanglement to form denser networks and improves compressive strength [61]. By contrast, carbon nanotube/graphene nanoplatelet-reinforced CS/polyvinyl alcohol/SA networks achieve elevated compressive moduli of 0.16–2.2 MPa [27]. Notably, enzymatically crosslinked silk fibroin/carboxymethyl CS (SF/CMCS) dual-network (DN) hydrogels stabilized via ethanol-induced β-folding reach the mechanical range of cancellous bone (compression modulus = 828.8 kPa, G′ = 88 kPa), maintain structural stability for 56 days (<9% mass loss), and provide sustained mechanical support for osteogenesis [60]. In addition, semi-interpenetrating composite gels (CG) fabricated from short-chain CS exhibit excellent mechanical properties and biocompatibility. These gels encapsulate ASA for sustained release over 14 days, thereby promoting PDLSC proliferation and osteogenic differentiation [62].

Collectively, mechanical adaptive optimization resolves the structural instability and mechanical mismatch of conventional CS scaffolds under dynamic oral environments, meeting the mechanical support requirements of load-bearing periodontal defects. Even so, a major trade-off persists within current scaffold optimization approaches: excessive crosslinking and high-concentration reinforcing dopants reduce scaffold degradability, leading to asynchronous scaffold degradation and new bone formation and ultimately impairing long-term bone tissue remodeling.

## 3. Core Regenerative Mechanisms of Functionalized CS-Based Hydrogels

### 3.1. Immune Microenvironment Remodeling Mechanism

**Figure 2:**
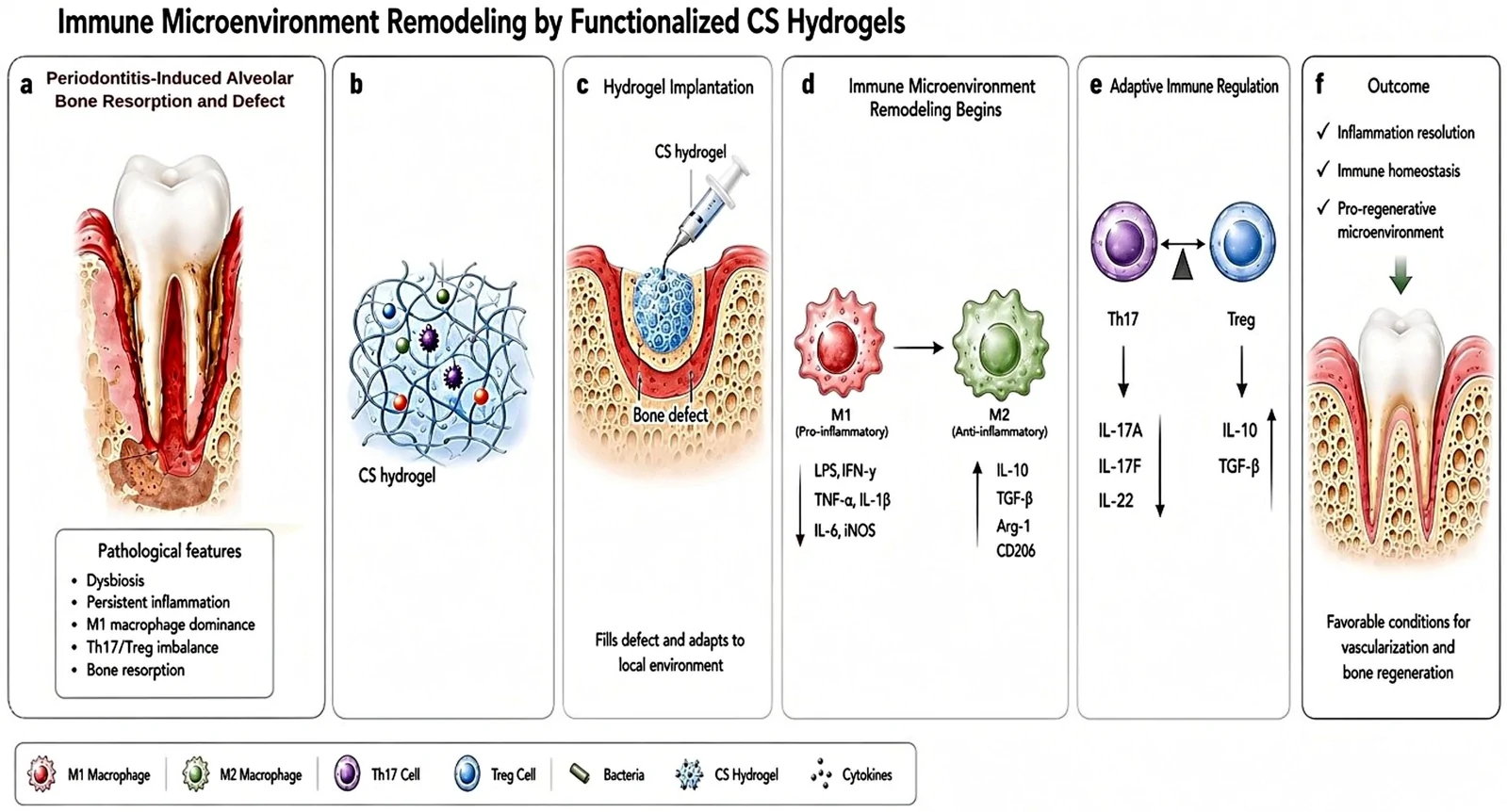
Schematic illustration of immune microenvironment modulation by a functionalized CS hydrogel. (**a**) Periodontitis-induced alveolar bone defect. (**b**) Composite scaffold loaded with multiple bioactive molecules. (**c**) Implantation of the functionalized scaffold into the bone defect site. (**d**,**e**) Immunomodulatory responses within the bone defect microenvironment. (**f**) Scaffold-mediated alveolar bone regeneration and periodontal tissue repair.

CS-based hydrogels act as active immunoregulatory platforms to modulate both innate and adaptive immune responses within periodontal defects. They achieve precise immune tuning by dynamically regulating the function of antigen-presenting cells (APCs), among which macrophages and DCs serve as the primary cellular targets. By modulating APC activation, phagocytic capacity, and cytokine secretion, CS-based hydrogels maintain immune homeostasis: they suppress pathological inflammation without compromising host defense, while concurrently facilitating tissue repair [63,64] (Figure 2). Numerous CS-based hydrogel systems—including CS/hyaluronic acid (HA), carboxymethyl CS (CMCS)/gelatin, and CS/β-glycerophosphate hydrogels—have been demonstrated to drive macrophage phenotypic switching from the pro-inflammatory M1 subtype toward the anti-inflammatory M2 phenotype [65,66,67]. Mechanistically, the timing of macrophage activation is critical for bone formation. Studies investigating macrophage behavior during bone regeneration show that M1-dominant phases correlate with osteogenic inhibition, whereas the subsequent shift to an M2 phenotype coincides with vascularization and osteogenesis [68,69]. Existing evidence thus validates immunomodulation as a key design objective, though the optimal magnitude and timing of such modulation remain context-dependent.

Beyond macrophage-centered regulation, CS-based hydrogels also alter lymphoid cell function. CS/poly(trimethylammonium chloride); hydrogels not only promote M2 macrophage polarization but also trigger the conversion of pro-inflammatory Th17 cells into immunosuppressive regulatory T cells (Tregs) in a rat full-thickness skin wound model [70]. Direct evidence demonstrating that quaternized or sulfated CS coordinates dendritic cell and macrophage–T cell crosstalk remains limited, and relevant supporting data originate from non-periodontal models. Quaternized CS promotes M2 polarization via the PI3K-Akt signaling pathway in vitro and attenuates inflammation in a rat subcutaneous infection model [22]. Likewise, chemically modified CS derivatives (e.g., quaternized CS and sulfated CS) modulate DC activation and fine-tune macrophage–T cell crosstalk. This cascade elevates the secretion of anti-inflammatory cytokines (e.g., IL-10), alleviates periodontal inflammation, and accelerates alveolar bone regeneration [12,71]. Furthermore, pH-responsive CS derivatives (CMCS, thiolated CS) exhibit tunable cationic density and charge-switching behavior under acidic inflammatory conditions. These properties enable dynamic adsorption and spatiotemporally controlled release of immunomodulatory agents (e.g., IL-4, IL-10) to achieve localized immune regulation [49].

Although the core immunoregulatory pathways of CS hydrogels have been verified, most mechanistic investigations rely on simplified single-cell in vitro models. The mechanisms governing immune cell crosstalk within the complex periodontal microenvironment—where bacterial infection, inflammation, and mechanical stimulation coexist—remain poorly characterized. Further work is required to dissect these integrated immunomodulatory networks under this pathological context. Longitudinal studies tracking immune phenotype, bacterial burden, and tissue formation within the same periodontal model will help establish stronger mechanistic associations.

### 3.2. Antibacterial and Anti-Inflammatory Synergistic Mechanism

CS-based materials couple antibacterial activity with inflammatory regulation through direct interactions with bacteria, localized delivery of antimicrobial agents, and modulation of host immune responses [70,72]. Protonated amino groups along CS molecular chains generate strong electrostatic interactions with anionic components of bacterial cell membranes (e.g., lipopolysaccharides and teichoic acids). These interactions disrupt membrane integrity, increase membrane permeability, and ultimately induce lysis and death of periodontal pathogens such as *P. gingivalis* and *F. nucleatum*, thereby eliminating bacterial infection [48,73]. The antimicrobial activity and spectrum of CS-based hydrogels can be further enhanced via structural modification or the incorporation of exogenous antibacterial agents. For example, CS hydrogels loaded with antimicrobial peptide Nal-P-113 and polydopamine nanoparticles (PDNPs) achieve > 99% inhibition of multispecies periodontal biofilm-forming bacteria (e.g., *F. nucleatum* and *Streptococcus gordonii*) under in vitro conditions [14]. As a representative example, black phosphorus-incorporated CS hydrogels ameliorate pathological lesions in diabetic periodontitis by tuning host iron homeostasis, driving macrophage polarization toward the anti-inflammatory M2 phenotype, and suppressing aberrant inflammatory responses [74].

Accumulating evidence confirms that functional CS-based composite hydrogels exhibit robust antibacterial and immunomodulatory efficacy in both in vitro and in vivo periodontitis models [50,74]. Nevertheless, available in vivo proof of their antibacterial effects against periodontitis is largely restricted to rodent models. Representative CS-based hydrogels tested in rat periodontitis models include: a multi-crosslinked system loaded with silver nanoparticle (AgNP)–lipid complexes [43], copper sulfide (CuS) nanoparticle-incorporated carboxymethyl and sulfonated CS [50], methacrylated CS blended with CuS nanoparticles [75], and CS/β-glycerophosphate hydrogels containing minocycline-loaded ZIF-8 nanoparticles [29]. These studies also confirm that metal-based nanoparticles and their released metal ions exert size- and concentration-dependent cytotoxicity. For instance, AgNPs smaller than 20 nm more readily induce cytotoxicity, and high concentrations of leached metal ions may trigger nonspecific cytotoxic damage to adjacent periodontal tissues and disrupt the homeostasis of oral commensal microbiota.

Such multi-mechanism synergistic antibacterial and anti-inflammatory systems endow CS hydrogels with distinct advantages over single-function antibacterial materials. However, most antibacterial assessments are conducted within simplified in vitro bacterial environments. Data regarding their stability in inflammatory exudate and biofilm microenvironments remain scarce, and in situ validation under clinically relevant pathological conditions is lacking.

### 3.3. Osteogenic Differentiation Induction Mechanism

CS-based hydrogels promote osteogenic regeneration via both physical and molecular pathways. Structurally, biomimetic porous scaffolds provide stable adhesion sites for osteogenic stem cells and regulate their morphological differentiation. At the molecular level, CS materials activate the Wnt3a/β-catenin and ERK1/2 osteogenic signaling pathways, upregulate osteogenic markers (Runx2, ALP, OCN), and facilitate osteogenic differentiation of BMSCs and PDLSCs [76]. Additional modification strategies further improve the bone remodeling, antibacterial, and anti-inflammatory properties of CS hydrogels, optimizing periodontal regeneration outcomes. For example, doping with strontium and copper ions inhibits osteoclast activation by modulating the RANKL/OPG ratio, reducing inflammatory bone resorption while amplifying osteogenic repair [77]. Injectable dual-network (DN) hydrogels composed of sulfated CS and oxidized HA display bifunctional characteristics: sulfated CS provides localized antibacterial activity, while the composite matrix robustly supports cell proliferation, osteogenic differentiation, angiogenesis, and periodontal ligament–bone integration [78]. CS hydrogels embedded with honeycomb-structured MnO_2_ nanozymes and BMP-2-derived peptides (BHM) enable spatiotemporally coordinated cascade regulation of hemostasis, antibacterial defense, anti-inflammatory responses, and osteogenic differentiation, orchestrating sequential events for ordered periodontal regeneration [79]. Hollow carboxymethyl CS/SA/CaCO_3_ composite scaffolds enhance structural stability and promote blood clot formation; sustained Ca^2+^ release recruits PDLSCs and MC3T3-E1 cells, upregulates core osteogenic markers (ALP, Runx2, OCN), and directs cell commitment toward the osteoblastic lineage [7]. Furthermore, multi-crosslinked CS-Pluronic F407-poly(N-isopropylacrylamide) (CS-P407-PNIPAM) hydrogels encapsulating AgNPs and CQ-ML effectively suppress *P. gingivalis* proliferation in a rat periodontitis model. Separately, pH-responsive CS/β-GP composite hydrogels loaded with minocycline hydrochloride release antibiotics in a pH-dependent manner to eradicate periodontal pathogens including *P. gingivalis* and *Aggregatibacter actinomycetemcomitans* (*A. actinomycetemcomitans*). Meanwhile, these hydrogels suppress NLRP3 inflammasome activation by inhibiting caspase-1 cleavage and subsequent IL-1β maturation. These dual therapeutic effects reduce ROS accumulation, mitigate local inflammation, and promote periodontal tissue regeneration [29].

Despite these advances, current investigations mainly focus on individual signaling pathways in CS hydrogel-mediated osteogenesis. Systematic crosstalk between immune regulation and osteogenesis remains underexplored, and the core molecular targets bridging inflammation resolution and bone formation have not been clearly defined.

### 3.4. Angiogenesis-Osteogenesis Coupling Mechanism

**Figure 3:**
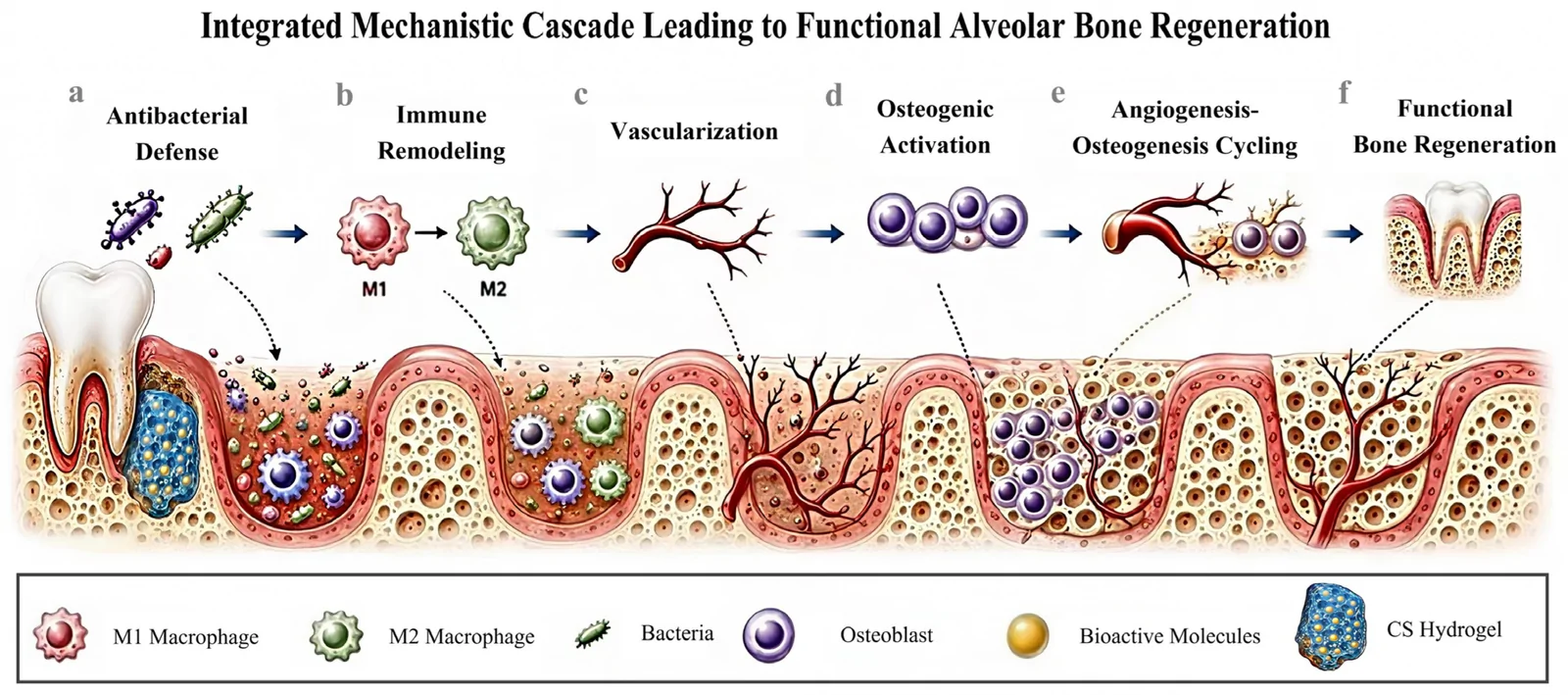
Schematic illustration of local tissue responses following scaffold implantation. (**a**) Local antibacterial defense. (**b**) Immune modulation. (**c**) Tissue vascularization. (**d**) Osteoblast activation. (**e**) Angiogenesis–osteogenesis coupling. (**f**) Functional bone regeneration.

Angiogenesis serves as a prerequisite for bone regeneration, supplying oxygen and nutrients required for new bone mineralization. Expression of VEGF—a key pro-angiogenic factor—is primarily governed by the hypoxia-inducible factor (HIF) signaling pathway. Under hypoxic conditions, HIF-1α is stabilized, accumulates in the cytoplasm, translocates into the nucleus, dimerizes with HIF-1β, and binds to hypoxia-response elements (HREs) within the VEGF promoter region, thereby upregulating VEGF transcription and initiating neovascularization [80]. CS-based hydrogels support angiogenesis through sustained delivery of pro-angiogenic factors including VEGF and EPO [36,53,81] (Figure 3). For instance, injectable CS composite hydrogels co-delivering VEGF and dental pulp stem cell (DPSC)-derived exosomes synergistically promote VEGF-mediated endothelial tube formation and DPSC-exosome-driven osteogenic differentiation of pre-osteoblasts [81]. Injectable thermosensitive hydrogels designed for sequential release deliver ASA at early stages to suppress acute inflammation, followed by EPO to stimulate endothelial cell proliferation and migration, establishing a pro-regenerative microenvironment conducive to bone healing [24]. EPO-loaded CS/β-GP/gelatin thermosensitive hydrogels increase new bone volume and mineral density in maxillary sinus floor elevation models [36]. CS–polycyclodextrin sponges also act as promising sustained-release platforms for VEGF to stimulate endothelial cell activity for bone regeneration [82].

Moreover, the antioxidant and immunomodulatory properties of CS hydrogels reduce ROS accumulation, alleviate tissue hypoxia, and indirectly facilitate angiogenesis [70]. Similarly, CS hydrogels co-loaded with gallic acid and zinc ions (Zn^2+^) enhance angiogenesis via dual mechanisms: suppressing local inflammation and reinforcing M2 macrophage polarization [83,84]. CS-based thermosensitive injectable hydrogels also promote vascularization by modulating adaptive immunity: they drive DC and T-cell differentiation toward anti-inflammatory phenotypes, relieving immune-mediated suppression of vessel growth [12,85]. Notably, a multifunctional CS composite hydrogel incorporating copper- and strontium-doped mesoporous bioactive glass and loaded with BMP-2 concurrently activates angiogenic signaling pathways (e.g., HIF-1α/VEGF) via controlled release of Cu^2+^/Sr^2+^ ions and robustly stimulates osteogenic differentiation [78].

The pro-angiogenic capacity of CS hydrogels has been widely demonstrated; however, the precise timing and dose coordination governing angiogenesis–osteogenesis coupling remains unclear. Excessive vascular proliferation may trigger fibrous hyperplasia and impair bone mineralization, highlighting the need for optimized coupled regulatory strategies.

### 3.5. Coupling Between Inflammation and Material Design

Within the inflammatory microenvironment, the antibacterial, immunomodulatory, osteogenic, and pro-angiogenic functions of biomaterials interact in a highly interconnected manner, often limiting the maximum biological performance of the materials. Three categories of synergistic effects are supported by current evidence.

Persistent inflammation and oxidative stress impose specific design requirements for CS-based biomaterials. Periodontitis is characterized by sustained oxidative stress [86], which may alter crosslinks formed by hydrolysable or oxidation-sensitive chemistries. Degradation of CS-based constructs may transiently reduce mesenchymal stem cell viability at high concentrations of released glucosamine [87]. Representative design strategies include incorporating ROS-scavenging moieties so the matrix consumes oxidants [22,51], and selecting crosslinking chemistries that yield biocompatible degradation products.

Dysbiosis and polymicrobial biofilms compromise the efficacy of monotherapy antibacterial approaches. These challenges motivate the development of multifunctional CS-based systems, in which CS’s intrinsic cationic antibacterial activity is combined with antimicrobial payloads, biofilm-responsive release, or adjunctive mechanical debridement. Spatial compartmentalization within polymicrobial communities reduces antimicrobial efficacy relative to single-species cultures tested in non-oral models [88]. The inherent antibacterial activity of CS has mostly been validated using planktonic bacteria or immature biofilms. Although in vitro assays have reported >99% inhibition of multispecies biofilm-forming bacteria [14], these experiments employed defined laboratory consortia rather than mature clinical biofilms. CS-based antibacterial strategies are therefore best regarded as adjuncts to mechanical debridement. Sustained antimicrobial release cannot fundamentally eliminate the risk of drug resistance, because prolonged exposure to subinhibitory concentrations enables periodontal microorganisms to develop resistance [89,90].

Immunomodulation is also phase-dependent. Early inflammatory signaling supports host defense, while subsequent inflammation resolution promotes vascularization and osteogenesis [68]. Accordingly, materials that globally suppress inflammatory signaling throughout the entire healing process fail to recapitulate the temporal biological cascade of tissue repair. Sequential delivery systems, such as early ASA administration followed by EPO release [24], exemplify design principles derived from this phase-dependent behavior. Consistent with this strategy, a multilayered CS/alginate scaffold delivering IL-4 induced M1-to-M2 macrophage polarization and enhanced bone regeneration in non-oral bone defects.

## 4. Challenges and Future Research Strategies

### 4.1. Scientific Challenges

A major unresolved challenge lies in reconciling the mechanical stability and degradation kinetics of chitosan-based biomimetic scaffolds with the dynamic cascade of native bone regeneration. Under physiological humid conditions, weakened intermolecular interactions within chitosan impair scaffold mechanical integrity. Meanwhile, enzymatic hydrolysis-driven degradation frequently creates a mismatch between scaffold degradation rate and new bone formation kinetics, further compromising regenerative performance.

Another key limitation is the structural instability of chitosan hydrogels within the inflammatory microenvironment. Sustained inflammation and elevated ROS levels in periodontitis-associated bone defects disrupt hydrogel cross-linking and network architecture. This triggers premature scaffold collapse and loss of bioactivity, ultimately impairing long-term therapeutic efficacy.

The third challenge concerns insufficient precision and controllability of biomolecule delivery. Since the release of growth factors and therapeutic payloads from CS hydrogels is highly sensitive to microenvironmental cues (pH, ionic strength, enzymatic activity), such systems fail to meet the requirements for stage-specific, spatiotemporally controlled release needed for periodontal regenerative therapy.

The fourth challenge is achieving contour preservation and space maintenance in inflammatory periodontal bone defects. PCL/PVA/CS blended nanofiber membranes, injectable hydrogels, 3D-printed scaffolds, sandwich-structured CS/PCL/gelatin composite scaffolds, and CS-derived Janus hydrogel barrier membranes all exhibit favorable space-maintaining capacity [91,92,93]. For example, the hierarchically porous EPO-functionalized scaffold (DMPS-PDA-EPO) fabricated via 3D printing produced a 161.9% higher bone volume fraction and a 167% higher bone mineral density at 8 weeks after implantation into rat periodontal bone defects [94]. Even so, commercially available bone sponges, bone powders, and periosteal membranes still fail to satisfy the mechanical requirements for clinical use, restricting their application mainly to the repair of infrabony defects. These outcomes are appropriate for alveolar bone regeneration but do not establish periodontal regeneration, which requires coordinated restoration of alveolar bone, periodontal ligament and cementum. This distinction should remain explicit when comparing studies and assessing translational relevance.

### 4.2. Clinical Translation

Chitosan-based hydrogel scaffolds face multiple hurdles during translational development from bench to bedside. Some obstacles originate from the materials’ physicochemical properties and the microenvironment at the implantation site, and the reliability of available research evidence varies substantially. Successful clinical translation requires comprehensive consideration of sterilization compatibility, the establishment of standardized testing protocols to rigorously control batch-to-batch variability, validation and stabilization of all manufacturing unit operations, as well as preservation of defect space and functional stability of materials within the oral microenvironment.

### 4.3. Future Research Directions and Strategies

Improve mechanical strength and structural stability while retaining biodegradability by incorporating reinforcing agents (nano-bioactive ceramics, ECM-derived polymers, synthetic polyesters) via physical blending or covalent crosslinking. Construct biomimetic ECM-mimetic architectures to enhance bioactivity, cell recruitment, and tissue integration.

Integrate dual immunomodulatory and antimicrobial functions by loading cytokine modulators, redox-balancing agents, and targeted antimicrobial agents. Design rational loading strategies and spatiotemporal release profiles to guarantee bioavailability, sustain effective local concentrations, and reduce off-target toxicity.

Develop multilayered, porous, or stimuli-responsive delivery systems for sequential release of therapeutic agents, angiogenic factors (e.g., VEGF), and osteogenic factors (e.g., BMP-2). Incorporate micro/nanoscale topographic patterning to build biomimetic vascular scaffolds, promoting vascular network formation and improving the quality of regenerated bone.

Beyond bone scaffolds, chitosan-based biomaterials have also been fabricated into periosteum analogues, nanoparticles, sponges, and other constructs for periodontal tissue and oral bone regeneration. When used as bone defect fillers, scaffolds, nanoparticles, and sponges rely on matched mechanical properties and degradation kinetics to preserve spatial stability of defect sites under functional occlusal loading of teeth. Therefore, further optimization of mechanical performance and degradation behavior remains essential for chitosan-based bone filling materials.

## 5. Summary

As versatile natural biomaterial scaffolds, CS-based hydrogels possess unique intrinsic advantages for repairing periodontitis-associated alveolar bone defects. Rational biomimetic structural optimization, immunomodulatory and anti-inflammatory modification, construction of smart responsive release systems, and mechanically adaptive design effectively overcome the inherent limitations of pure CS hydrogels. These modifications enable the integration of antibacterial defense, inflammatory microenvironment remodeling, pro-angiogenic effects, and osteogenic regeneration. Nevertheless, current findings are limited to specific animal models and material formulations.

Multiple bottlenecks hinder clinical translation, including the challenging trade-off between mechanical properties and degradation kinetics, insufficient durability and stability of inflammatory modulation, poor spatiotemporal controllability of therapeutic functions, and the lack of standardized fabrication protocols and translational evaluation frameworks. The vast majority of studies remain at the preclinical stage, with very limited evidence from human studies. Furthermore, most investigations primarily assess alveolar bone formation rather than the coordinated regeneration of alveolar bone, periodontal ligament, and cementum.

Accordingly, future research should prioritize the following objectives:

(1) Establish explicit dose–effect relationships between CS chemical architecture and drug release profiles;
(2) Develop inflammatory disease models that faithfully recapitulate the pathological hallmarks of periodontitis;
(3) Improve the mechanical performance of bone-filling materials and optimize the barrier function of membrane materials to maintain the structural stability of bone defect sites;
(4) Establish manufacturing workflows that preserve consistent material performance after sterilization and long-term storage.

With continued advances in dynamic biomimetic architectures, stimuli-responsive platforms, and personalized three-dimensional (3D) printing techniques, CS-based hydrogels hold great promise as next-generation scaffold candidates for treating periodontitis-associated bone defects.

## Figures and Tables

**Table 1:** Systematic comparison of four design strategies for CS-based hydrogels in alveolar bone regeneration.

Optimization Strategy	Classification Basis	Core Technical Measures	Advantages	Existing Limitations	Applicable Periodontal Scenarios	References
**Structural biomimetic design**	Spatial architecture: pore network, anisotropy and matrix composition	Macroporous and nanoporous regulation; double-network construction; layered or gradient fabrication; nanomaterial composite reinforcement	Improves pore connectivity and cell infiltration; mimics native bone extracellular matrix; stabilizes scaffold geometry; supports cell adhesion and tissue ingrowth	Structural optimization alone provides no antibacterial or anti-inflammatory function; some nanocomposites degrade poorly; layered fabrication is complex and poorly reproducible	Large irregular alveolar bone defects; defects requiring multi-tissue interface reconstruction	[18,19,20]
**Inflammatory immune regulation**	Host immune response: macrophage, dendritic cell and T cell phenotype	Loading anti-inflammatory drugs, immunomodulators or bioactive ions; regulating macrophage and T cell polarization	Resolve pathological inflammation and build a pro-regenerative immune environment	Single immune modulation provides no mechanical support; burst release of immunomodulators may be cytotoxic; the duration of immune modulation is usually short	Defects with active or chronic inflammation; infectious bone resorption	[12,21,22]
**Stimuli-responsive controlled release**	Temporal control: matching release to the healing sequence	pH-, temperature- or ROS-responsive modification; staged sequential release of anti-inflammatory and osteogenic factors	Align drug delivery with the inflammation–repair sequence and reduce off-target exposure	Responsiveness is unstable in a heterogeneous inflammatory microenvironment; coordinating several factors is difficult	Dynamic inflammatory defects; defects requiring staged repair	[23,24,25]
**Mechanical microenvironment adaptation**	Load transfer and structural stability under cyclic loading	Polymer blending; covalent or physical crosslinking; bioactive ceramic doping; dynamic network design	Increases strength and toughness; match the modulus of alveolar cancellous bone; improves resistance to masticatory loading	High-strength modification often slows degradation; dynamic mechanical adaptation remains limited; mechanical and degradation behavior are rarely coupled by design	Load-bearing periodontal defects; defects in occlusal load-bearing regions	[26,27]

## Data Availability

No new data were created or analyzed in this study. Data sharing is not applicable to this article.
